# Effect of the Composition of CAD/CAM Composite Blocks on Mechanical Properties

**DOI:** 10.1155/2018/4893143

**Published:** 2018-10-23

**Authors:** Rasha A. Alamoush, Nick Silikas, Nesreen A. Salim, Suhad Al-Nasrawi, Julian D. Satterthwaite

**Affiliations:** ^1^School of Dentistry, The University of Manchester, Manchester, UK; ^2^Prosthodontic Department, University of Jordan, Amman, Jordan; ^3^Faculty of Dentistry, University of Kufa, Najaf, Iraq

## Abstract

The aim of this study was to evaluate the effect of the composition of CAD/CAM blocks on their mechanical properties. Nine different CAD/CAM blocks, enamel and dentine, were tested. Sixteen samples of each material were separated for Vickers microhardness test (n=6, 5 readings per specimen), nanohardness test (n=6, 5 readings per specimen), filler weight (n=3), and SEM imaging (n=1). Data were statistically analysed using one-way ANOVA. Vita Mark II ceramic showed significantly higher values of hardness (in both nano- and microscale) and elastic modulus (6.83 GPa, 502 kg/mm^2^, and 47.7 GPa), respectively, than other materials. CAD/CAM composite blocks showed comparable values of hardness and elastic modulus to those of dentine but lower than those of enamel and ceramics. SEM images highlighted different filler-matrix microstructure of CAD/CAM composite blocks. It was concluded that (1) hardness and elastic moduli are positively correlated with ceramic filler percentage and microstructure and (2) CAD/CAM composite materials have comparable hardness and elastic moduli to tooth structure.

## 1. Introduction

Alternative aesthetic restorations have been introduced, as the digital technologies of CAD/CAM systems have been developed. Ceramic and composite have been used as indirect restorations using CAD/CAM systems as promising restorations [1]. In addition, PEEK has been proposed for CAD/CAM prosthodontics applications [2].

Ceramic has favourable properties for use as an indirect restorative material; it is a very biocompatible and strong material [1–3]. However, it is very stiff, hard, and brittle; these properties affect its clinical performance, durability, and machinability. In terms of their hardness and stiffness, ceramics are considered highly abrasive. This affects the material performance in two aspects: first, clinically, ceramic might cause opposing enamel wear and roughness [4, 5]. Second, it is difficult to machine; for instance, in CAD/CAM systems ceramic hardness causes milling tool damage over time; also it takes longer to mill compared with composite [6, 7]. The other main disadvantage of ceramics is brittleness. Again, it affects the material in two main aspects: First, ceramic clinical durability is highly related to their brittleness; most ceramic restorations fail because of crack propagation due to the material brittleness [8]. Second, in terms of machinability, ceramic might chip or crack during processing. Hence it is difficult to manufacture even with CAD/CAM systems [9].

Over the years, indirect composite restorations have improved in relation to their mechanical properties in different ways: alteration of the composition (monomer resins, initiation systems); incorporation of high percentage filler particles; and polymerization modes (using high temperature and pressure for polymerization) [10, 11]. These have improved both tensile and compressive strength, hardness, elastic modulus [12], and wear resistance [13, 14]. CAD/CAM technology allows for many of these alterations in manufacturing to result in improved indirect composite restorations.

CAD/CAM composite has the following main advantages compared to ceramic: it has less hardness and stiffness, so the opposing enamel exhibits less wear clinically. In addition, it is easily fabricated and repaired. It is also less brittle [15]. Consequently, less catastrophic failure is expected as well as less chipping and crack introduction during manufacturing [9]. In addition, they are more compatible with milling machine and exhibit better marginal quality [9, 10, 16].

Different formulations have been introduced recently with different material classifications such as ceramic-like materials, polymer infiltrated ceramics, CAD/CAM resin based blocks, or nanoceramics [3, 17]. CAD/CAM composites can be classified based on their microstructural geometry into two main types, resin with dispersed fillers and polymer infiltrated ceramic networks [18].

PEEK has favourable mechanical properties [19]. It has similar tensile properties to those of bone, enamel, and dentine [20]. Therefore, it has been proposed for use in fixed [21] and removable prostheses [22]. Further investigation of CAD/CAM composites in many aspects such as mechanical properties, bonding, and biocompatibility is highly needed. Most importantly, their mechanical properties such as flexural strength, flexural modulus, modulus of resilience, and hardness that can predict the material clinical success and performance are important to be evaluated [23–25].

In the view of limited research on CAD/CAM composite blocks and the need to evaluate their clinical success and performance, this study aimed to test the mechanical properties (hardness, elastic modulus, and microstructure) of different CAD/CAM blocks and compare them to ceramic, enamel, and dentine using two indentation techniques (nanoindentation and Vickers hardness). The null hypotheses were that (1) there is no difference in the tested mechanical properties between materials and (2) the mechanical properties of the tested materials will not be affected by the their composition.

## 2. Materials and Methods

### 2.1. Study Design

Nine different CAD/CAM blocks were tested (n=16 each group). Enamel and dentine discs were prepared from extracted wisdom teeth (n=12, each group). Samples of each material were allocated into two groups: Vickers microhardness test (n=6) and nanohardness test (n=6). In addition 4 samples (of each CAD/CAM block) were used for filler weight test (n=3) and SEM imaging (n=1). The microhardness was measured by means of a Vickers indenter tester (FM-700, Future Tech Corp., Japan). The test parameters were with load of 300 g and 20 s dwell time. Nanoindentation measurements (elastic modulus, hardness) were undertaken using a nanoindenter (M3 Nanovea, Nanovea Co., CA, USA) equipped with a Berkovich three-sided pyramidal diamond tip. The machine was set for the chosen parameters: load of 20 g and pause of 20 s. Thirty indentations on 6 samples (5 for each) were made for each material for each test. SEM images at 1000x and 5000x magnifications were obtained to assess filler-matrix microstructure of hybrid ceramics. Data were statistically analysed using one-way ANOVA.

### 2.2. Materials and Sample Preparation

The nine CAD/CAM blocks used in this study were resin composite CAD/CAM block (Lava Ultimate, Shofu, Cerasmart, Brilliant Crios, Grandio Blocs); polymer infiltrated ceramic network (PICN) ceramic block (Enamic); pure PEEK (Ceramill PEEK); ceramic filled PEEK (Dentokeep); and feldspathic ceramic block (Vitablocs Mark II). Enamel and dentine discs were prepared from extracted wisdom teeth. A list of materials studied, with details of filler percentage and polymer, is given in Table 1.

Six specimens of each of the 11 materials were prepared (9 CAD/CAM blocks, enamel and dentin). Each CAD/CAM block was sectioned into rectangular bars of 2 mm thickness using a diamond blade (MK 303, MK diamond, CA, USA) mounted on a saw (Isomet 1000 Precision Cutter; Buehler Co, IL, USA) under constant water irrigation (ISO 6872:2008) [26]. Discs of 2 mm of enamel and dentine were prepared from extracted wisdom teeth from young adults (ethical approval was granted by NHS, Health Research Authority, London, Harrow Research Ethics Committee (15/LO/1545)) and disinfected with 20 ml of 5% sodium hypochlorite for 10 min (301696S, BDH Chemicals Ltd., Poole, BH15, England) and then wrapped in cotton gauze saturated with physiologic saline (59300C, Dulbecco's Phosphate Buffered Saline, Sigma-Aldrich Inc., St. Louis, USA) and kept at 4°C to be prepared and tested within a week.

All specimens were wet ground and polished with a lapping machine (MetaServ 250, Buehler Co, IL, USA) with a series of silicon carbide papers (SiC) and paper disks P320, P500, P1200, P2400, and P4000-grit (Buehler Co, Illinois, USA) under water cooling and then polished with 0.25 um diamond suspension (Meta Di Supreme, Buehler Co, IL, USA) and cleaned in an ultrasonic bath (Ultrasonic Cleaning System, L&R Co, NJ, USA) with distilled water for 5 min. The specimens were stored dry for 24 hr at room temperature.

### 2.3. Nanoindentation

Elastic modulus and hardness measurements (nanoindentation measurements) were obtained using a nanoindenter (M3 Nanovea, Nanovea, Co., CA, USA) equipped with a Berkovich three-sided pyramidal diamond tip which was used with indenter cone angle 130.54 and elastic modulus of 1140 GPa.

Calibration indents were made on a fused silica sample with an elastic modulus of 71.3 GPa and hardness of 8.9 GPa. The machine was set for the chosen parameters, load of 20 g, and pause of 20 s. Poisson's ratio for all tested materials was assumed to be 0.3. Thirty indentations were undertaken (5 for each sample) for each material at room temperature. The maximum load applied by the nanoindenter to examine the specimens was 20 g. The machine calculated the elastic modulus (E) and nanohardness (H) by using the generated force-displacement curves from nanoindentation testing, based on the Oliver-Pharr method [27], using the following equations, respectively:

(1) $$\begin{array}{c} E=\frac{1}{2}\times \frac{\surd \pi }{\surd A}\times \frac{dh}{dP} \end{array}$$

where A is the projected contact area; dh is the change in depth; dP is the difference in load.

(2) $$\begin{array}{c} H=\frac{Pmax}{A} \end{array}$$

where A is the projected contact area; Pmax is the maximum load.

### 2.4. Vickers Microhardness

Surface microhardness was measured by means of a Vickers indenter tester (FM-700, Future Tech Corp., Japan) under a 300 g loading and 20 s dwell time. For each indentation, both diagonals (D1, D2) were measured using the microscope. Five indents were undertaken for each sample in a straight line. The distance between the indentations was calculated by multiplying the average indentation diagonal length by four (4*∗*D) to ensure sufficient distance between the indentations. Five indentations were undertaken on each specimen and the hardness values were averaged. Thirty determinations on 6 samples were made for each material. The machine then automatically calculated the corresponding hardness value and presented it as VHN. Vickers microhardness can also be calculated using the following equation [28]:

(3) $$\begin{array}{c} VHN=1.854\;\;\frac{P}{D^{2}} \end{array}$$

where P is the applied load in kg and D is the indentation diagonal length in mm.

### 2.5. Filler Content

The mass percentage of inorganic filler content of the CAD/CAM blocks was measured by elimination of the organic part of the CAD/CAM blocks by heating at a constant temperature (ash technique) in accordance with ISO 1172:1996 [29]. Thermogravimetric analysis (TGA) is an alternative method to measure the filler content and is possibly more accurate. Three samples of each material (n=3) were kept in an electric furnace (Programat EP 5000, Ivoclar Vivadent, Liechtenstein, Austria) set at 625°C for 30 min and then cooled in a desiccator. The samples were then weighed to an accuracy of 0.01 mg using a calibrated electronic analytical balance (Ohaus Analytical Plus, Ohaus Corporation, USA). The percentage of inorganic fillers by weight was then determined using the following equation:

(4) $$\begin{array}{c} Filler\;\;weight\%=\left[\;\left(\;100-\left(\;\frac{\left(m1-m2\right)}{m1}\;\right)\;\right)\times 100\%\text{)}\;\right] \end{array}$$

with m1 being the mass before heating and m2 the mass after heating and cooling.

### 2.6. Microstructure

The surfaces of the specimens were wet polished using SiC paper P600 up to P4000 and diamond solutions of 9, 3, 1, and 0.25 *μ*m and subsequently ultrasonically cleaned with acetone for 5 min. The specimens were dried, mounted on aluminium stubs, and sputter-coated with carbon. The surface of each specimen was examined using a scanning electron microscope (SEM; FEI Quanta200, OH, USA) and SEM images at 1000x and 5000x magnifications at 10 kV were obtained.

### 2.7. Statistical Analysis

All results were tested using Levene's test for homogeneity of variance (P <0.05), following the assumption of equal variances. Equal variances were confirmed (P > 0.05); hence the Bonferroni post hoc test was used to determine the differences in the mechanical properties (hardness, elastic modulus, and microstructure). For the filler weight percentage measurement, Bland and Altman test was used to compare the measured values with the manufacturers' values where a minimal variation was detected with high level of reproducibility.

## 3. Results

**Figure 1:**
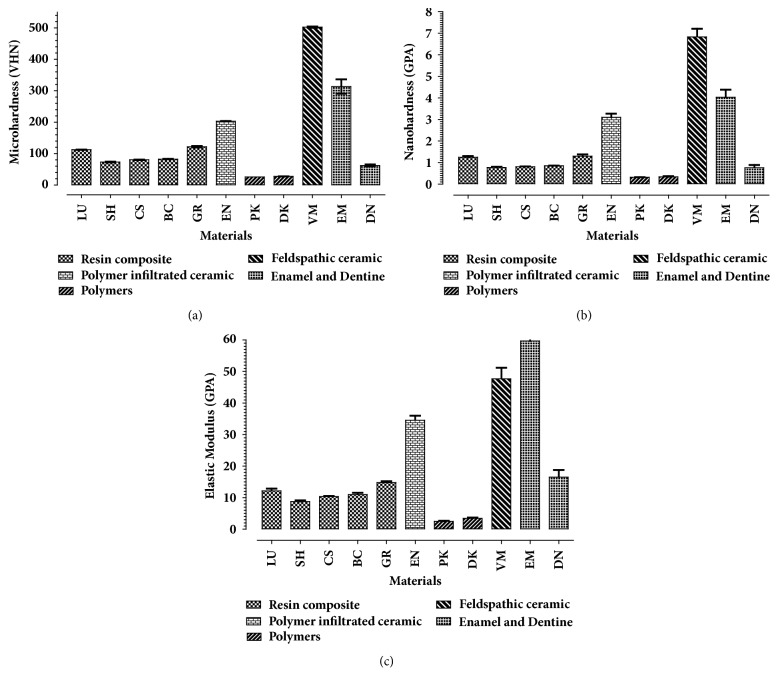
A bar chart showing the mean (a) Vickers surface microhardness (standard deviation); (b) nanohardness; (c) elastic modulus of eleven tested materials; resin composite CAD/CAM blocks (Lava Ultimate, Shofu, Cerasmart, Brilliant Crios, Grandio Blocs); polymer infiltrated ceramic network (PICN) ceramic block (Enamic); pure PEEK (Ceramill PEEK); ceramic filled PEEK (Dentokeep); and feldspathic ceramic block (Vitablocs Mark II); enamel and dentine.

Mean microhardness, nanohardness, and elastic modulus for all tested materials are shown in Table 2 and Figure 1. A statistically significant difference in the means of microhardness, nanohardness, and elastic modulus between the tested materials was revealed.

The values of nanohardness ranged from 0.31 (SD.008) GPa for pure PEEK to 3.1 (SD 0.17) GPa for Vita Mark II ceramic. The values of microhardness ranged from 25.7 (SD 0.05) Kg/mm^2^ for pure PEEK to 502.4 (SD 2.28) Kg/mm^2^ for Vita Mark II ceramic. The values of elastic modulus ranged from 2.53 (SD 0.15) GPa for pure PEEK to 59.7 (SD 13) GPa for enamel.

**Figure 2:**
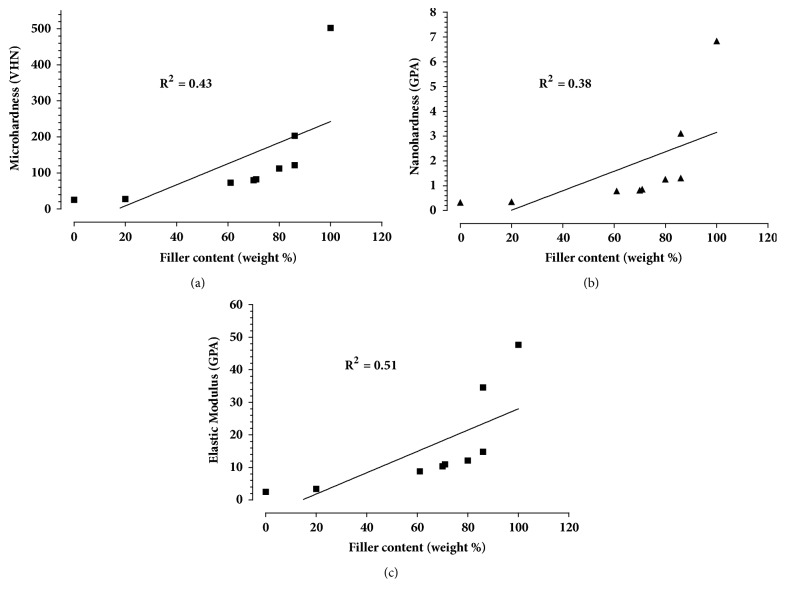
A scatter plot showing a positive correlation and linear regression between filler weight percentage and (a) microhardness (VHN), R^2^=0.43,* P*=0.05, (b) nanohardness (GPA), R^2^=0.38,* P*=0.07, and (c) elastic modulus (GPA), R^2^=0.51,* P*=0.03.

The measured and manufacturers' filler percentages by weight are presented in Table 2. Measured values of filler content ranged from 100% (SD 0.0) weight for Vitablocs Mark II to 0.00 for pure PEEK. The measured filler percentage by weight was compared to the manufacturers' filler percentage. A Bland and Altman test shows minimal variation between results and high reproducibility. Elastic modulus, microhardness, and nanohardness were correlated with filler weight percentage and the results showed a positive correlation with linear regression: for filler weight percentage and microhardness (VHN), R^2^=0.43, P=0.05; for nanohardness (GPa), R^2^=0.38, P=0.07; for elastic modulus (GPa), R^2^=0.51, P=0.03 (Figure 2). In addition, the nanohardness and elastic modulus values were highly correlated where R^2^=0.93.

**Figure 3:**
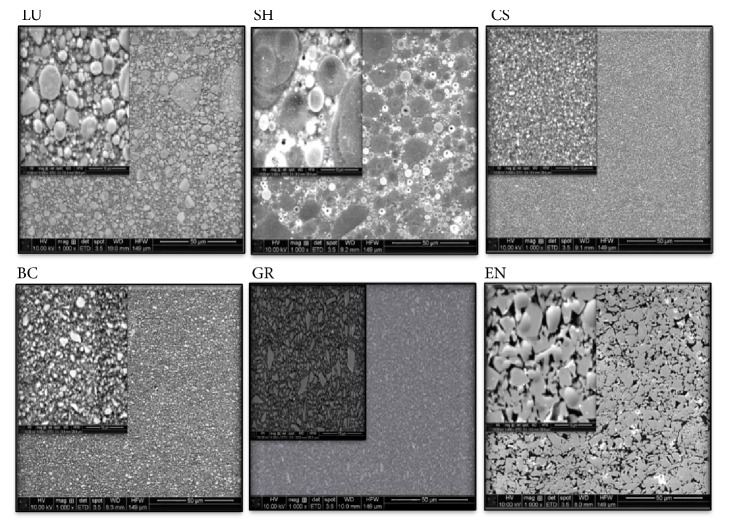
SEM images of CAD/CAM blocks of six tested materials at 5000x and 1000x magnifications at 10 Kv, detector; ETD, spot size 3.5, WD; 10 mm.

SEM images showed different microstructures of the tested CAD/CAM composite blocks (Figure 3). SH contained two varieties of spherical particles, CS contained relatively large and small and uniformly distributed particles, BC contained small and uniformly distributed particles, GR contained two varieties of particle, large and small irregularly shaped particles, LU contained a wide range of particle sizes, and EN exhibited a dense ceramic network structure with resin matrix. It can be noticed that CAD/CAM composite blocks had versatile microstructural constituents as well as variable filler weight percentages.

## 4. Discussion

The results of the present study show that the tested materials were significantly different in their mechanical properties (microhardness, nanohardness, and elastic modulus). CAD/CAM composite was significantly different from ceramics, enamel and dentine. Consequently, both null hypotheses were rejected.

It was noticed that CAD/CAM composite blocks had different microstructure as well as variable filler weight percentages and hence differences in the tested mechanical properties. However, it seems that the filler percentages have a more considerable role in these properties than do the microstructural constituents. In fact, PICN (EN) might be an exception as it exhibited higher values of hardness and elastic modulus compared to other CAD/CAM composite blocks, which could be attributed to the manufacturing technique of polymer and ceramics networking.

Of the tested materials, Vita Mark II ceramic had the highest value of hardness (in both nano- and microscale) and elastic modulus, and it was significantly higher than the values of enamel and dentine rendering VM as very hard and stiff material. This might be considered a disadvantage in terms of machinability and durability [9, 25]. Enamic showed higher hardness and elastic modulus values compared to other resin composite CAD/CAM blocks, which might be attributed to the robust microstructural geometry of PICN as compared to other resin composite CAD/CAM blocks which are basically a resin with dispersed ceramic fillers. The PICN VHN, nanohardness, and elastic modulus were in between the enamel and dentine values but closer to enamel values. The tested resin composite CAD/CAM blocks were closer in their characteristics to dentine rather than enamel.

The values of the elastic moduli of PICN (Enamic) 34.56 GPa and resin composite CAD/CAM blocks are close to the values of enamel and dentine when compared to CAD/CAM ceramic which is very hard and stiff. This means they are closer to the tooth structure stiffness. Nonfilled and low filled ones (PEEK and Dentokeep) had lower hardness and elastic modulus values. These results were comparable with similar studies [10, 30–32].

The aim of any dental restorative material is to have similar characteristics to that of the toot structure [25, 33]. Hence, resin ceramic combination in a network structure exhibits the positive characteristics of ceramics and resin [24]. This material has low rigidity, hardness, and stiffness but high flexibility and fracture toughness [34, 35]. Resin with dispersed ceramic fillers has good fracture and wear resistance and high compressive strength [36].

PEEK showed favourable mechanical properties [19]. It has similar tensile properties to those of bone, enamel, and dentine [20]. Therefore, it has been proposed for use in fixed [21] and removable prostheses [22]. Although PEEK is increasingly used in fixed prosthodontics, the values of hardness and elastic modulus in this study were considerably low. However, for low percentage ceramic filled PEEK, the values were higher than pure PEEK; this is obviously attributed to the ceramic fillers. Low filled PEEK and PEEK have comparable hardness and stiffness values with PMMA. Hence, they might be a good choice for long-term restorations [32].

Nanoindentation is a well-documented method to measure the mechanical properties of both dental materials and teeth [37]. This test was used in this study to measure the elastic modulus as well as the hardness values of the tested materials at nanoscale. Using the microhardness test usually creates a relatively large indentation size of about 100 *μ*m, while nanoindentation allows applying load as low as 30 mN and indentation size less than 5 *μ*m [38].

Nanoindentation might be a more precise method to investigate materials with microstructural constituents, such as microfilled or nanofilled composites [39]. However, it has limitations, mainly that the test is very sensitive to thermal changes and mechanical vibration and acoustic noise [40]. Also the indenter tip size in relation to the filler particle size [41] and the maximum load used is relevant; i.e., if the indenter tip size or load were too small it would not provide sufficient information about the bulk material properties [39]. However, the appropriate indenter size along with appropriate load will provide sufficient information about the material properties. Also, there is no microscope linked to the Nanovea machine, so the location of the tested point can only be determined by naked eyes.

Vickers microhardness test is a versatile method that can be used to measure hardness for a wide range of materials and easy to employ. The main advantage is that the indentation geometry does not change due to different loads or different tested materials. However, there is an operator subjective variation as the indentation surface area is determined according to the average length of both diagonals (d) which can be determined microscopically by naked eyes [42].

Although both tests give hardness values, their values cannot be directly or simply compared due to the different testing mechanisms such as indenter type, test settings, and loading force. In nanoindentation the hardness depends on the applied load and indentation depth which is being measured as a function of the applied load (P) and the size of the contact area of indentation which depends on the geometry of the indenter [40]. However, in Vickers microhardness test, the hardness relies on the surface area of a square-shaped indentation. The indentation surface area is determined according to the average length of both diagonals (d) of the square-shaped indentation [28].

## 5. Conclusions

Within the limitations of this study, the following conclusions can be drawn:

- The hardness and elastic moduli are positively correlated to ceramic filler percentage and microstructure.
- CAD/CAM composite materials have comparable hardness and elastic moduli to tooth structure.
- CAD/CAM composites combine ceramic good strength with composite lower hardness. But further in vivo work is warranted to determine its clinical relevance and serviceability.

## Figures and Tables

**Table 1:** Materials tested and manufacturer's information.

Material type	Materials * (Code)*	*Composition by weight*		Manufacturer
		Filler	polymer	
Resin composite CAD CAM blocks	Lava™- Ultimate (LU)	80% silica and zirconia nano particles	20% ( Bis-GMA, UDMA, Bis-EMA, TEGDMA)	3M™ESPE™ USA
	Shofu (SH)	61% Silica-based glass and silica	UDMA+TEGDMA	Shofu
	Cerasmart (CS)	71% Silica and barium glass nanoparticles	Bis-MEPP, UDMA, DMA	GC dental products, Europe
	BRILLIANT Crios (BC)	70% of glass and amorphous silica	Cross-linked methacrylates (Bis-GMA, Bis-EMA, TEGDMA)	COLTENE, Switzerland
	Grandio Blocs (GR)	86% Nanohybrid fillers	14% UDMA+ DMA	VOCO GmbH

Polymer infiltrated ceramic network (PICN) ceramic	Vita Enamic (EN)	86% ceramic	14% UDMA+TEGDMA	Vita Zahnfabrik, Germany

Pure PEEK	Ceramill PEEK (PE)	0	100% PEEK	Juvora, UK

Ceramic filled PEEK	Dentokeep (DK)	20% TiO_2_	80% PEEK	Nt-trading Germany

Feldspathic ceramic block	Vitablocs Mark II (VM)	Fine-particle feldspar ceramic	0	Vita Zahnfabrik, Germany

**Table 2:** Mean (SD) Vickers microhardness, nanohardness, elastic modulus, and the measured and manufacturers' filler percentages by weight (wt%) for all tested materials. Values with the same superscript letters per column represent nonsignificant statistical difference for each individual property (*α*=0.05).

Material type	Material *(code)*	Microhardness *(Kg/mm*^*2*^)	Nanohardness *(GPa) *	Elastic Modulus *(GPa) *	Manufacturers' Filler *(wt*%)	measured filler *(wt*%)
Resin composite CAD/CAM blocks	Lava™- Ultimate (LU)	112.6 (0.44)^c^	1.25 (0.05)^c^	12.14 (0.76)^c^	80	74.8(0.1)
	Shofu (SH)	73.12 (1.04)^d,f^	0.775 (031)^d^	8.79 (0.35)^c,d,e^	61	63 (0.02)
	Cerasmart (CS)	80.06 (0.76)^d^	0.81(0.006)^d^	10.36 (0.17)^c,d,e^	70	66.1(0.2)
	BRILLIANT Crios (BC)	82.61(0.49)^d^	0.85 (0.008)^d^	10.98 (0.6)^c,e^	71	70.1(0.05)
	Grandio Blocs (GR)	121.8(2.1)^c^	1.3(0.08)^c^	14.8(0.4)^c^	86	84.6(0.01)

Polymer infiltrated ceramic network (PICN) ceramic	Vita Enamic (EN)	203.1(0.43)^b^	3.1 (0.17)^b^	34.56 (1.4)^b^	86	85.1(0.1)

Pure PEEK	Ceramill PEEK (PE)	25.7 (0.05)^g^	0.317(0.008)^e^	2.53 (0.15)^d^	0	.00(0)

Ceramic filled PEEK	Dentokeep (DK)	27.74 (0.19)^g^	0.34(0.03)^e^	3.43 (0.29)^d,e^	20	27.5(0.06)

Feldspathic ceramic block	Vitablocs Mark II (VM)	502.4 (2.28)^a^	6.83 (0.379)^a^	47.7 (3.47)^a^	100	100(0)

Enamel	EM	313.3 (22.7)^h^	4.03(0.35)^f^	59.7(13)^f^	-	-

Dentine	DN	62.3 (3.3)^f^	0.76( 0.13)^d^	16.5(2.3)^c^	-	-

## Data Availability

The data used to support the findings of this study are included within the article.
